# The effect of 12 weeks *Anethum graveolens* (dill) on metabolic markers in patients with metabolic syndrome; a randomized double blind controlled trial

**DOI:** 10.1186/2008-2231-20-47

**Published:** 2012-10-04

**Authors:** Masoume Mansouri, Neda Nayebi, Abasali keshtkar, Shirin Hasani-Ranjbar, Eghbal Taheri, Bagher Larijani

**Affiliations:** 1 Endocrinology & metabolism research centre, Tehran university of medical sciences, Tehran, Iran; 2 Golestan gastroenterology and hepatology research center, Golestan university of medical sciences, Gorgan, Iran

**Keywords:** Anethum graveolens, Dill, Metabolic syndrome, Hyperlipidemia, Clinical trial, Abdominal obesity

## Abstract

**Background:**

The clustering of metabolic abnormalities defined as metabolic syndrome is now both a public health and a clinical problem .While interest in herbal medicine has greatly increased, lack of human evidence to support efficacies shown in animals does exist. This clinical trial study designed to investigate whether herbal medicine, *Anethum graveolens* (dill) extract, could improve metabolic components in patients with metabolic syndrome.

**Methods:**

A double-blind, randomized, placebo-controlled trial using a parallel design was conducted. 24 subjects who had metabolic syndrome diagnostic criteria (update of ATP III) were randomly assigned to either dill extract (n = 12) or placebo (n = 12) for 3 months.

**Results:**

Across lipid component of metabolic syndrome, no significant differences in triglyceride (TG) concentration and high density lipoprotein cholesterol were seen between the two groups. However TG improved significantly from baseline (*257.0 vs. 201.5p = 0.01*) with dill treatment but such a significant effect was not observed in placebo group. Moreover, no significant differences in waist circumference, blood pressure and fasting blood sugar were seen between two groups after 3 months follow up period.

**Conclusion:**

In this small clinical trial in patients with metabolic syndrome, 12 weeks of dill extract treatment had a beneficial effect in terms of reducing TG from baseline. However dill treatment was not associated with a significant improvement in metabolic syndrome related markers compared to control group. Larger studies might be required to prove the efficacy and safety of long-term administration of dill to resolve metabolic syndrome components.

## Introduction

The clustering of metabolic abnormalities defined as metabolic syndrome does occur in one quarter of the world’s adults. People with metabolic syndrome are three times as likely to have a heart attack or stroke compared with people without the syndrome and have a five-fold greater risk of developing type 2 diabetes [1].

While metabolic syndrome causes the two global epidemics of type 2 diabetes and cardiovascular disease (CVD), it overpowers any effort to identify and treat those individuals with metabolic syndrome early, so that new and effective treatment methods may prevent the development of diabetes and/or CVD [1].

Although, complementary and alternative therapies have long been used in the Eastern world, interest to herbal medicine along with the isolation and standardization of herbal constituents has greatly increased worldwide and would open a new approach for novel therapeutic and more effective agents to treat or prevent diseases such as diabetes mellitus [2].

*Anethum graveolens L*. known as dill is a sparse looking plant with feathery leaves and tiny yellow flowers growing in the Mediterranean region, Europe, central and southern Asia and is widely cultured in south eastern region of Iran [3]. The metabolic syndrome is a complex of interrelated risk factors for CVD and diabetes [4]. In an urban population of Tehran MONICA project using the Adult Treatment Panel III (ATPIII) criteria showed that the crude prevalence rate of the metabolic syndrome was 29.9 %, meaning that more than one fourth of the population are at risk of CVD and diabetes [5]. As a result, the metabolic syndrome is now both a public health and a clinical problem urging investigations on new methods for its treatment.

While the use of herbal medicine is gaining considerable recognition and popularity worldwide, a lack of medical evidence to support its therapeutic efficacy does exist and a few herbs have been clinically studied [6]. As a folk remedy *Anethum graveolens* or dill is considered for some gastrointestinal ailments such as flatulence, indigestion, stomachache [7] and in traditional Iranian medicine (TIM) has been used as carminative, antispasmodic, sedative, lactagogue, diuretic and for hyperlipidemia [8].

Pharmacological effects of dill such as anti-inflammatory [9], antimicrobial [10-12] or antibacterial [13,14] , antihyperlipidaemic , antihypercholesterolaemic [15-17] , antioxidative and hypoglycemic [16-18] activities have been shown. To date, the role of dill extract in the improvement of metabolic syndrome parameters has not been investigated. However there have been two human randomized clinical trials evaluating the lipid lowering effect of dill extract. Unlike most studies carried on animals, a few human studies showed this herbal treatment has no significant reducing effect in hyperlipidemic patients [19,20].

It is worth mentioning that in human studies, hyperlipidemic patients have received a patented compound named *Anthum* which consists mostly of *Anethum graveolens* (68%) and less of *Cichorium intybus*, *Fumaria parviflora* and *Citrus sp*[21].

Due to the not existence enough human studies, we designed a double blind randomized, placebo-controlled trial to evaluate whether purred extracted dill could safely improve metabolic parameters in patients with metabolic syndrome.

## Material and methods

### Participants

A total of 110 ambulatory, community-dwelling Tehranian men and women volunteers between the ages of 18 and 50 were considered for initial screening. They were invited through the flyers distributed throughout the Shariati Hospital wards particular places where visible to public from January to December 2009. All volunteers were interviewed by attending physician regarding to life style, present and past medical history and using any medication or supplements.

If volunteers met initial eligibility criteria through the first interview, underwent physical exams and primary laboratory test to participate in this study. Metabolic syndrome were defined based on the AHA/NHLBI update of Adult Treatment Panel (ATPIII) criteria [22] consisting of

1) Elevated waist circumference >88 cm (female), >102 cm (male)

2) Reduced HDL cholesterol < 50 mg/dl (female), <40 mg/dl (male)

3) Elevated triglyceride ≥ 150 mg/dl

4) Elevated blood pressure ≥130/85 mmHg

5) Elevated fasting blood glucose ≥ 100 mg/dl

Exclusion criteria included any history of clinical cardiovascular disease (myocardial infarction, angina, stroke, heart failure), diabetes mellitus, systemic, neurologic, psychiatric and endocrine diseases, liver function test abnormalities (aspartate aminotransferase or alanine aminotransferase levels > 3 times upper limit of normal), renal insufficiency (creatinine levels > 2.5 mg/dl) women who were pregnant, breastfed or have a pregnancy plan for the next 6 months and use of any medications, dietary supplements and herbals except of analgesics within 30 days prior to screening. Participants with a diagnosis of hypothyroidism could be included only if their level of TSH was within the normal range while they were receiving at least 3 months of a stable dose of thyroid replacement. Finally a total of 24 patients who had ≥3 of the metabolic syndrome diagnostic criteria and other eligibility criteria entered the study. Eighty six of participants were excluded because of the following reasons; in first lab results we noticed that many participants didn’t fulfill the metabolic syndrome criteria. Some of them were on diet and less inclined to participate in wash out period and a few were newly diagnosed of type 2 diabetes.

### Study protocol

A double-blind, randomized, placebo-controlled trial using a parallel design with a 3-month follow up period, was established. A permuted-balanced-block randomization was employed to generate the random assignment of 24 subjects by order of entry into two different treatment groups. Each group received either *Anethum graveolens* extract (AGE) or placebo, one pill (600 mg) per day for 3 months. Subjects were visited during four time’s period: basal, first month, second month and third month. Twelve- hour fasting blood samples were obtained before each visiting session. During each visit anthropometric measurements and vital signs were assessed and they were asked questions about unexpected adverse events.

Body weight was assessed with a standard scale while they were lightly dressed and barefoot. Height was measured using a wall-mounted stadiometer. The body mass index (BMI) was measured by dividing the weight (kg) to height (m^2^). Waist circumference (WC cm) defined as the minimal abdominal circumference between the xiphoid process and iliac crest. Left hand blood pressure was measured by using standard mercury sphygmomanometer in the sitting position. The blood pressure values were confirmed by a second measurement after 5 minutes.

All study personnel were blinded to treatment assignment. All participants were told to maintain their usual dietary habits (diet stability was assessed and verified by analyzing all dietary recalls taken during each study visit by a registered dietitian). Adherence to study medication was assessed by pill count at each visit. Protocol of the study has been approved (Code: E-0046) by both the institutional review board and ethics committee of endocrinology and metabolic research institute (EMRI) of Tehran University of Medical Sciences. After giving required information about the details of the study program, all participants assigned written consent form.

### Preparation *of dill extract*

Dried aerial parts of *Anethum graveolens* including leaves and steams were purchased locally from herbal markets. The plant was identified by herbaria’**s** expert of pharmacology department of Tehran University of Medical Sciences (TUMS). The hydro alcoholic extract of dried dill was prepared in the food and drug research laboratory of EMRI using following procedure. For the preparation of the hydro alcoholic extract, 100 gram of the dried grounded plant was suspended in 400 mL double distilled water- ethanol (2:1; v/v). The extract was filtered and the filtrate was evaporated to dryness with a rotatory vacuum evaporator. Then the dried powder (600 mg) was put into empty gelatin capsules by pharmaceutical company. More over the other gelatin capsule containing rice starch powder in the same color employed as a placebo. The toxicity assessment of hydroalcoholic extract of oral *Anethum graveolens* in animal was evaluated by Arbabi et al [23]. According to this study doses less than 50 mg/kg could be considered as no observable adverse effect level (NOAEL) and could be used in the future clinical investigations.

### Assessment of adverse events

After initiation of the study, drug-related adverse effects were assessed by using questionnaire, physical examination and laboratory tests. The physician filled out a copy of the healthy questionnaire based on interview which was about changes in their general health since their last visit and physical examination of the patient. Besides vital sign monitoring (temperature, blood pressure, pulse rate) cardio respiratory and abdominal examination were also performed per visiting session (in each visit). Potentially adverse reaction on kidney and liver function were evaluated by measuring serum creatinine, electrolytes, liver enzyme, bilirubin (total and direct) and albumin at baseline and each visit. Drug related hematologic reaction was assessed by measuring the serum levels of all blood cells. Shifts from normal range and clinically and statistically significant deviation from baseline values of laboratory parameters were recorded as adverse events at each visit.

### Biochemical analysis

White and red blood cell counts and hemoglobin levels were measured using a Multi hematologic Analysis System (Sismex, Japan). The following biochemical parameters were measured in the EMRC blood analysis laboratory using an automatic analysis system (Autoanalyzer; Hitachi Ltd, Tokyo, Japan) with enzymatic kits (Pars Azmoon, Iran): serum triglyceride (TG mg/dl), total cholesterol (TC mg/dl), high-density lipoprotein cholesterol(HDL-C mg/dl), low-density lipoprotein cholesterol (LDL-C mg/dl), fasting blood glucose (FBS mg/dl), alkaline phosphatase (ALP IU/L), alanine aminotranferease *(*ALT IU/L*)* and aspartate aminotransferase *(*AST IU/L*)*, creatinine (mg/dl), blood urea nitrogen (mg/dl) and albumin (g/dl). All inter-assay calculated coefficients of variation were within the normal range of enzymatic kits data sheets.

### Statistical analysis

Results are expressed as means ± SD. Significance of differences was evaluated using the SPSS statistical program package (SPSS; 16, Inc, USA) and defined at 0.05 level of confidence. Normality assumption of variables was assessed by Kolmogorov*-*Smirnov test and then the comparison between groups mean differences at baseline and each month was performed by student's t test. Within groups mean changes from baseline to final values at 4, 8 and 12 weeks in component of metabolic syndrome and other variable were analyzed by using paired t test.

## Results

**Figure 1:**
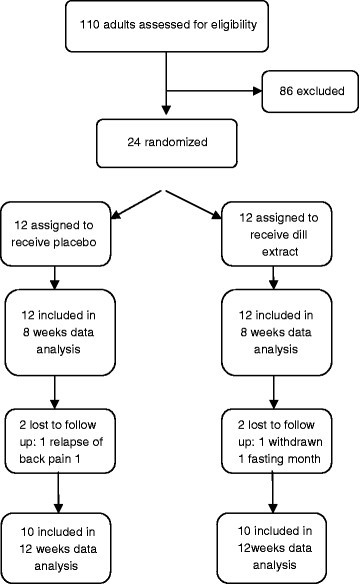
Flow of the trial.

Over an 11-month period, 110 healthy individuals were screened for the study, and 24 were eligible based on entry criteria. 20 of 24 participants completed all study-related visits. Thus the twelve weeks data analysis was performed in 20 subjects who completed the study (10 subjects in each group).Patient recruitment and screening process for trial is shown in Figure 1.

The demographics and baseline characteristics of the study subjects in the two groups were similar and there were no significant differences between the two groups for metabolic syndrome parameters including WC, HDL-C, BP, FBS and TG (Table 1).

**Table 1:** Baseline characteristics of Study Participants

**Variables**	**Placebo (n = 12)**	**Dill extract (n = 12)**	**P value**
Age(yr)	36.8(9.6)	38.2(8.2)	0.7
Sex Male n (%)	7(58.3)	6(50.0)	0.5
Weight(kg)	97.2(17.5)	103.1(26)	0.5
BMI(kg/m^2^)	37.8(7.4)	36.6(8.1)	0.7
WC (cm)	107.8(13.7)	110.8(14.3)	0.6
SBP (mm Hg)	121.0(12.9)	121.6(17.7)	0.9
DBP (mm Hg)	84.0(9.4)	81.3(10.3)	0.5
WBC	6.9(2.0)	6.2(1.69)	0.3
PLT	278.6(93.4)	220.2(77.1)	0.1
FBS (mg/dl)	101.0(11.7)	105.6(12.3)	0.3
Serum creatinine (mg/dl)	0.9(0.1)	0.9(0.21)	0.4
TG(mg/dl)	252.8(111.7)	257.0(124.5)	0.8
TC(mg/dl)	215.7(23.01)	210.9(24.0)	0.6
LDL-C(mg/dl)	122.1(18.5)	116.9(17.3)	0.4
HDL-C(mg/dl)	37.9(4.0)	39.4(6.8)	0.5
AST (IU/L)	28.0(13.09)	21.00(7.23)	0.1
ALT (IU/L)	39.6(31.6)	22.27(14.5)	0.1
ALP(IU/L)	190.0(56.3)	187.8(56.9)	0.9

Value are presented as mean(SD).BMI, body mass index ; WC, waist circumference; SBP, systolic blood pressure; DBP, diastolic blood pressure; WBC, White blood cells*;* FBS, fasting blood sugar; TG, triglyceride; TC, total cholesterol; HDL, high-density lipoprotein; LDL, low-density lipoprotein; AST, aspartate transaminase; ALT, alanine transaminase; ALP, alkaline phosphatase.

### Changes in metabolic syndrome parameters after 8 weeks

The 8 weeks data analysis was performed for 24 participants. Compared to baseline values no significant changes in metabolic syndrome components were found after 8 weeks in dill treatment group (Table 2). Moreover blood analysis showed no significant changes in markers for kidney and liver function as determined by serum creatinine and liver enzymes. In addition, placebo using for 8 weeks didn’t significantly affect metabolic syndrome parameters and mentioned markers.

**Table 2:** Changes in metabolic syndrome parameters and other variables from baseline to 8–week follow-up

**Variable**	**Placebo Group (n = 12)**			**Dill Extract Group (n = 12)**			**Dill vs placebo**
	**After 8 weeks**	**Mean change**	**P**	**After 8 weeks**	**Mean change**	**p**	**P**
Weight(kg)	96.3(20.1)	−0.9	0.1	100.9(27.0)	−2.2	0.1	0.6
BMI(kg/m^2^)	35.2(6.9)	−2.6	0.2	36.1(7.5)	−0.5	0.2	0.9
WC (cm)	107.7(13.6)	−0.1	0.7	109.0(14.8)	−1.8	0.7	0.8
SBP (mm Hg)	117.7 (17.9)	−3.3	0.5	116.8(14.8)	−4.8	0.5	0.6
DBP (mm Hg)	80.5 (9.9)	−3.5	0.7	77.8(12.0)	−3.5	0.2	0.8
WBC	6.8(2.2)	−0.1	0.8	7.0(1.4)	0.8	0.8	0.8
PLT	268.4(75.8)	−10.2	0.9	250.9(65.4)	30.7	0.9	0.6
FBS (mg/dl)	95.7(12.1)	−5.3	0.3	105.8(17.6)	0.2	0.3	0.1
Serum creatinine (mg/dl)	0.9(0.2)	0.0	0.6	0.9(0.1)	−0.02	0.6	0.6
TG(mg/dl)	230.1(57.9)	−22.7	0.1	232.3(100.6)	−24.7	0.1	0.9
TC(mg/dl)	215.3(19.1)	−0.4	0.5	206.6(28.2)	−4.3	0.8	0.3
LDL- C(mg/dl)	122.5(12.9)	0.4	0.7	113.1(14.7)	−3.6	0.4	0.1
HDL-C(mg/dl)	39.8(8.2)	1.9	0.4	38.5(6.9)	−0.7	0.5	0.7
AST (IU/L)	20.2(6.9)	−7.8	0.2	20.7(8.9)	−0.3	0.2	0.8
ALT (IU/L)	26.8(19.5)	−12.8	0.2	28.0(23.1)	6.7	0.2	0.5
ALP (IU/L)	164.8(40.9)	−25.2	0.1	191.57(54.5)	3.77	0.1	0.4

Values are expressed as mean (SD). BMI, body mass index ;WC, waist circumference; SBP, systolic blood pressure; DBP, diastolic blood pressure; WBC, White blood cells*;* FBS, fasting blood sugar; TG, triglyceride; TC, total cholesterol; HDL, high-density lipoprotein; LDL, low-density lipoprotein; AST, aspartate transaminase; ALT, alanine transaminase; ALP, alkaline phosphatase.

### Changes in metabolic markers after 12 weeks

Regarding to vital signs (pulse rate, respiratory rate) and physical exams, both groups didn’t show any significant changes compared to baseline (Data not shown). There was no significant change in Body Mass Index (BMI) and WC and blood pressure at the end of 12 weeks of treatment and two groups were not different from each other. In addition there were no statistically significant changes in dietary intakes (calories) between baseline and month 3 for each of the treatment groups as assessed by analyzing dietary recalls taken at each study visit, suggesting that diet remained stable during the treatment period in both groups. After 3 months of dill extract or placebo consumption, mean fasting blood glucose didn’t differ between two groups. Across lipid component of metabolic syndrome, no significant differences in fasting TG concentration and HDL-C were seen between the two groups. However TG improved significantly from baseline (*257.0 vs. 201.5p = 0.01*) with dill treatment but such a significant effect was not observed in placebo group. Moreover LDL-C was also not significantly different between the two groups (Table 3).

**Table 3:** Changes in metabolic syndrome parameters from baseline to 12-weeks follow-up

**Variable**	**Placebo Group (n = 10)**			** Dill Extract Group (n = 10)**			***Dill vs placebo***
	**After 12 weeks**	**Mean change**	**P**	**After 12 weeks**	**Mean change**	**p**	**P**
Weight(kg)	101.4(17.7)	4.2	0.2	100.7(28.9)	−2.4	0.7	0.9
BMI(kg/m^2^)	36.9(7.4)	−0.9	0.2	35.8(8.1)	−0.8	0.8	0.7
WC (cm)	111.4(13.5)	3.6	0.8	108.8(15.2)	−2	0.7	0.7
SBP (mm Hg)	113.4(11.7)	−7.6	0.2	116.0(18.9)	−5.6	0.3	0.7
DBP (mm Hg)	76.8(7.03)	−7.2	0.2	77.8(11.7)	−3.5	0.1	0.8
WBC	5.7(1)	−1.2	0.04	6.8(1.1))	0.6	0.4	0.3
PLT	289(59.9)	10.4	0.7	210.9(37.3)	−9.3	0.7	0.004
FBS (mg/dl)	100(15.8)	−1.3	0.6	103.0(11.9)	−2.6	0.2	0.6
Serum creatinine (mg/dl)	1.0(0.1)	0.1	0.6	0.93(0.2)	0.03	0.7	0.8
TG(mg/dl)	197.1(86.1)	−55.7	0.2	201.5(115.5)	−55.44	<0.01	0.9
TC (mg/dl)	209.1(23.8)	−6.6	0.3	196.7(26.9)	−14.2	0.2	0.3
LDL-C (mg/dl)	124.4(15.8)	2.3	0.8	109.2(16.1)	−6.7	0.6	0.1
HDL-C (mg/dl)	39.0(4.7)	1.1	0.9	36.1(7.3)	−3.3	0.2	0.3
AST (IU/L)	26.6(9.5)	−1.4	0.3	23.9(11.3)	2.9	0.3	0.1
ALT (IU/L)	37.7(13.3)	−1.9	0.3	27.9(27.0)	5.6	0.4	0.9
ALP (IU/L)	179.6(56.0)	−10.4	0.2	175.2(49.3)	−12.6	<0.01	0.5

Value are presented as mean(SD).BMI, body mass index; WC, waist circumference ; SBP, systolic blood pressure; DBP, diastolic blood pressure; WBC, White blood cells*;* FBS, fasting blood sugar;TG, triglyceride; TC, total cholesterol; HDL, high-density lipoprotein; LDL, low-density lipoprotein; AST, aspartate transaminase; ALT, alanine transaminase;ALP, alkaline phosphatase.

### Safety and tolerability

Safety was assessed by physical examination, evaluation of vital signs, and various laboratory measurements. There were no changes in clinical laboratory assessment including liver enzymes, total bilirubin, albumin, creatinine, and electrolytes among any of the treatment groups during follow up period. In addition, no apparent differences were observed in the safety parameters including vital signs, results of physical examination and hematologic tests in both groups. The only changes in hematologic tests were a significant trend for WBC to decrease in placebo group after 12 weeks, compared to baseline. Moreover a significant difference was observed in platelet level in dill group compared to placebo. In general both capsules were well tolerated with only few subjects had complained about the smell of the dill capsules, but they had continued the study to the end.

## Discussion

The present double blind randomized controlled trial examined whether the AGE or dill extract is capable to improve the lipid profile and other metabolic syndrome determinants. To our knowledge, this is the first study to demonstrate an effect for a pure dill extract on subjects with metabolic syndrome. Lipid disturbance is a prominent feature of metabolic syndrome. Although many studies have reported beneficial effect of dill treatment on lipid profile but most of these investigations have been conducted on animals [15-17,24]. The findings of the present study showed that dill extract resulted in significant reduction in TG levels from baseline to week 12. However serum TG was not significantly different in dill group compared with placebo. The result of our study in agreement with the conclusion of previous human studies reporting dill consumption has no effect on lipid profile in hyperlipidemic patients [19,20]. But these results are in contrast to what has previously been reported by animal studies [15-17,24].The discrepancy in results may be attributed to several factors including differences in prescribed dosage, kind of dill consumption, non standardization of the dill manufacturing method and variation in the analysis method. One of the main differences between animal and human studies was prescribed dosage .Considering weight factor the prescribed dosage in animal study is many times more than that used in human studies. The results of animal studies indicate that AGE in a dose dependent manner reduces lipid profile components. It seems that additional human trials are needed to clarify the amount of dill to produce clinically meaningful effect on metabolic markers without inducing the serious adverse events. Another difference is that we used a hydro alcoholic pure extract of dill, however, in those former studies combination preparation of several herbs with 68 % of dill, patented as “*Anethum*” was investigated [21]. It is shown that plant density and harvest timing influences dill oil composition in a complimentary manner[25]. Moreover the lack of standardization in herbal preparation may affect the content of the active ingredient and its efficacy [26].

A part from those mentioned, we suggest that different analysis method could be another explanation for the conflicting results between studies. Some studies reported significant decrease in lipid profile in response to dill consuming used only within group’s analysis. Comparing baseline and after dill intervention we also found that TG level significantly decreased in dill group. While comparing after treatment TG measures between dill and placebo group we didn’t find any significant differences (Table 3). It also seems appropriate to consider that lipid lowering effect of dill may be due to its ability to interact with or potentiate the action of various antihyperlipidemic agents. In the other hand lipid reducing effect of dill might appear more efficient in combination with diet or other hypolipidemic agents .Thus more investigation are needed in this regard.

Abnormality in blood pressure and fasting blood sugar are other important features of metabolic syndrome. In the present study neither blood pressure measures nor fasting blood sugar were affected by dill treatment. Regarding blood pressure no previous studies have attempted to describe blood pressure response to dill consumption. It is worth mentioning that the mean blood pressure in our population was not high. It seems that the effect of dill treatment would be more evident and better evaluated if the patients had high initial blood pressure. In general, dill extract was well tolerated and no adverse reaction was observed. We have no explanation for mild decrease in WBC in placebo group and platelet differences between two groups. However these changes were in the normal range and were not clinically significant. The lack of finding efficient effect of dill extract on the feature of metabolic syndrome in this study might be partly related to small population available for this study.

The strict inclusion criteria and consequently carefully selected cases restricted our ability to find larger sample size. Indeed we excluded the patients with concomitant medication, to assess the pure effect of dill extract and to prevent interaction effect of other treatments. These two although are strength points of this study but limit the ability to provide adequate statistical power for documenting the results. The shortness of the length study period caused to not include more eligible patients. In addition, the variation of the components contributing the metabolic syndrome diagnosis could be another limitation. In other words, we considered at least three criteria for the metabolic syndrome definition to include patients in this study, but these criteria were different from one to other.

## Conclusion

In this small clinical trial in patients with metabolic syndrome, 12 weeks of dill extract treatment had a beneficial effect in terms of reducing TG from baseline. However dill treatment was not associated with a significant improvement in metabolic syndrome related markers compared to control group. Larger studies might be required to prove the efficacy and safety of long-term administration of dill extract to resolve metabolic syndrome components.

## Competing interests

None of the authors.

## Authors contribution

M M***;*** study researcher*,* interpreted the results, added data and their interpretation, wrote the second version of paper and revised the paper. N N; study researcher, proposal writer, A K; analyzed and interpretation the data. S H-R; Study designer, supervisor of conduction of the study and writing the paper. E T; co- study designer. B L; co- study designer. All authors have contributed to, seen and approved the manuscript*.*

## References

[B1] Alberti KG Zimmet P Shaw J Metabolic syndrome-a new world-wide definition. A Consensus Statement from the International Diabetes Federation Diabet Med 2006 23 5 469 480 10.1111/j.1464-5491.2006.01858.x 16681555

[B2] Hasani-Ranjbar S Larijani B Abdollahi M A systematic review of Iranian medicinal plants usefulin diabetes mellitus Arch Med Sci 2008 4 3 285 292

[B3] Yazdanparast R Bahramikia S Evaluation of the effect of Anethum graveolens L. crude extracts on serum lipids and lipoproteins profiles in hypercholesterolaemic rats DARU 2008 16 2 88 94

[B4] Alberti K Eckel R Grundy S Zimmet P Cleeman J Donato K Harmonizing the metabolic syndrome: a joint interim statement of the international diabetes federation task force on epidemiology and prevention; National heart, lung, and blood institute; American heart association; World heart federation; International atherosclerosis society; and International association for the study of obesity Circulation 2009 120 1640 1645 10.1161/CIRCULATIONAHA.109.192644 19805654

[B5] Fakhrzadeh H Ebrahimpour P Pourebrahim R Heshmat R Larijani B Metabolic Syndrome and its Associated Risk Factors in Healthy Adults: Apopulation-based study in Iran Metab Syndr Relat Disord 2006 4 1 28 34 10.1089/met.2006.4.28 18370767

[B6] Vuksan V Sievenpiper JL Koo VY Francis T Beljan-Zdravkovic U Xu Z American ginseng (Panax quinquefolius L) reduces postprandial glycemia in nondiabetic subjects and subjects with type 2 diabetes mellitus Arch Intern Med 2000 160 7 1009 1013 10.1001/archinte.160.7.1009 10761967

[B7] Heber D PDR for herbal medicines 2004 Montvale: NJ Thomson 650 651

[B8] Zargari A Medicinal Plants 1996 Tehran: Tehran University 528 531

[B9] Tuntipopipat S Muangnoi C Failla ML Anti-inflammatory activities of extracts of Thai spices and herbs with lipopolysaccharide-activated RAW 264.7 murine macrophages J Med Food 2009 12 6 1213 1220 10.1089/jmf.2009.1118 20041774

[B10] Delaquis PJ Stanich K Girard B Mazza G Antimicrobial activity of individual and mixed fractions of dill, cilantro, coriander and eucalyptus essential oils Int J Food Microbiol 2002 25; 74 1–2 101 109 10.1016/s0168-1605(01)00734-6 11929164

[B11] Jirovetz L Buchbauer G Stoyanova AS Georgiev EV Damianova ST Composition, quality control, and antimicrobial activity of the essential oil of long-time storeddill (anethum graveolens L.) seeds from Bulgaria J Agric Food Chem 2003 18; 51 13 3854 3857 10.1021/jf030004y 12797755

[B12] Singh G Kapoor IP Pandey SK Singh UK Singh RK Studies on essential oils: part 10; antibacterial activity of volatile oils of some spices Phytother Res 2002 16 7 680 682 10.1002/ptr.951 12410554

[B13] Kaur GJ Arora DS Antibacterial and phytochemical screening of anethum graveolens. foeniculum vulgare and trachyspermum ammi BMC Complement Altern Med 2009 9 30 10.1186/1472-6882-9-30 19656417 PMC2736926

[B14] Rafii F Shahverdi AR Comparison of essential oils from three plants for enhancement of antimicrobial activity of nitrofurantoin against enterobacteria Chemotherapy 2007 53 1 21 25 10.1159/000098246 17192709

[B15] Yazdanparast R Alavi M Antihyperlipidaemic and antihypercholesterolaemic effects of Anethum graveolens leaves after the removal of furocoumarins Cytobios 2001 105 410 185 191 11409638

[B16] Madani H Mahmoodabady NA Vahdati A Effects of hydroalcoholic exteract of anethum graveollens (dill) on plasma glucose and lipid levels in diabetes induced rats Iranian Journal of Diabetes and Lipid Disorders 2005 5 2 35 42

[B17] Bahramikia S Yazdanparast R Efficacy of different fractions of anethum graveolens leaves on serum lipoproteins and serum and liver oxidative status in experimentally induced hypercholesterolaemic rat models Am J Chin Med 2009 37 4 685 699 10.1142/S0192415X09007168 19655407

[B18] Panda S The effect of Anethum graveolens L. (dill) on corticosteroid induceddiabetes mellitus: involvement of thyroid hormones Phytother Res 2008 22 12 1695 1697 10.1002/ptr.2553 18814208

[B19] Kojuri J Vosoughi AR Akrami M Effects of anethum graveolens and garlic on lipid profile in hyperlipidemic patients Lipids Health Dis 2007 6 5 10.1186/1476-511X-6-5 17328819 PMC1821028

[B20] Mirzadeh A Fotouhi A Allaoddini F Yazdani K Arya A Asghari F Randomizeddouble blind, placebo-controlled trial of anethum, nicotin acid and clofibrate in patients with isolated hypertriglyceridaemia Iranian journal of diabetes and lipid disorders 2002 1 2 139 148

[B21] Cherahgali A Iranian National Formulary, Iranian Ministry of Health and Medical EducationTehran 2000

[B22] Grundy SM Metabolic syndrome scientific statement by the american heart association and the national heart, lung, and blood institute Arterioscler Thromb Vasc Biol 2005 25 11 2243 2244 10.1161/01.ATV.0000189155.75833.c7 16258150

[B23] Arbabi SB TaheriE BH Ziarati P Safety evaluation of oral anethum graveolens L total hydroalcoholic extract in mice Journal of Pharmaceutical & Health Sciences 2011 1 2 61 67

[B24] Hajhashemi V Abbasi N Hypolipidemic activity of anethum graveolens in rats Phytotherapy Research 2008 22 372 375 10.1002/ptr.2329 18058989

[B25] Callan NW Johnson DL Westcott MP Welty LE Herb and oil composition of dill (anethum graveolens L.): Effects of crop maturity and plant density Ind Crop Prod 2007 25 3 282 287 10.1016/j.indcrop.2006.12.007

[B26] Hasani-Ranjbar S Nayebi N Moradi L Mehri A Larijani B Abdollahi M The Efficacy and Safety of Herbal Medicines Used in the Treatment of Hyperlipidemia; A Systematic Review Curr Pharm Des 2010 16 2935 2947 20858178 10.2174/138161210793176464

